# Modified nucleotides may have enhanced early RNA catalysis

**DOI:** 10.1073/pnas.1809041117

**Published:** 2020-03-30

**Authors:** Steven K. Wolk, Wesley S. Mayfield, Amy D. Gelinas, David Astling, Jessica Guillot, Edward N. Brody, Nebojsa Janjic, Larry Gold

**Affiliations:** ^a^SomaLogic, Inc., Boulder, CO 80301

**Keywords:** RNA world, modified nucleotides, catalytic RNA

## Abstract

The modern version of the RNA World Hypothesis begins with activated ribonucleotides condensing (nonenzymatically) to make RNA molecules, some of which possess (perhaps slight) catalytic activity. We propose that noncanonical ribonucleotides, which would have been inevitable under prebiotic conditions, might decrease the RNA length required to have useful catalytic function by allowing short RNAs to possess a more versatile collection of folded motifs. We argue that modified versions of the standard bases, some with features that resemble cofactors, could have facilitated that first moment in which early RNA molecules with catalytic capability began their evolutionary path toward self-replication.

There remains a robust debate about the transition from a primordial soup of molecules to organisms with Darwinian descent, a moment that Carl Woese (1, 2) called the Darwinian Threshold. Many scientists carry the idea that the RNA World Hypothesis, an expression first coined by Gilbert (3), provides the framework for the earliest piece of what was likely a gradual transition from chemical soup to catalysts and genetics. The critical first pieces of the RNA World Hypothesis are simple: the early Earth was complex chemically, and at some moment, RNA molecules, made initially through nontemplated condensations of activated monomers (4, 5), assumed the diverse roles of templates for replication, catalysts/ribozymes to do that replication, facilitators of other critical steps (such as energy utilization), and eventually, the storage of genetic information.

Each of the stages of evolution—going from simple molecules, like cyanide, to more complex molecules, like nucleic acids, to polymers capable of complex functions and ultimately, to systems capable of catalysis and templated replication—represents distinct and poorly understood problems. Although these steps involve vastly different processes and undoubtedly involve progressively increasing molecular complexity, each also required some form of partitioning to allow molecules the chance to not lose each other in the vastness of the early Earth. For later steps, such as energy utilization and genetics, formation of membranes and eventually, cells could have created the needed molecular localization. For early chemical condensations, the partitions could have been small primordial pools, where proximity was facilitated by evaporation or the presence of rocks composed of useful minerals (4). It has also been proposed that an ice–water interface during colder periods may have served as a protocellular medium that, among other features, concentrated the reactant molecules to levels not easily achievable by other means (6).

In this paper, we have tried to focus only on one issue within this long chain of events: how is it possible to evolve catalytic RNAs that function as short molecules with enough catalytic power to bootstrap the development of those short, chemically derived RNAs into replicators/genetics? In other words, what were the earliest ribozymes in an RNA World, and how did they get their start? These huge questions are addressed, along with many others, in wonderful (but slightly dated) reviews of the RNA World Hypothesis (7, 8). Bernhardt (8) noted that “[c]atalysis is a relatively rare property of long RNA sequences only,” and he did note that some catalytic RNAs are short and that one might imagine that the environment of the early Earth could have facilitated RNA-mediated catalysis. Essentially, Bernhardt (8) said, “please do not worry about this problem.” Here, we have ignored his plea.

Scientists have imagined that RNAs randomly assembled at sufficient lengths are all one might need to achieve a transition to a stable RNA World. We argue that length is less important than catalytically competent shape, which we refer to as “globularity.” Globularity in RNAs (like the pockets within proteins that constitute the active sites, which we associate with protein *g*lobularity) as well as the availability of nucleobases with catalytic function could facilitate the emergence of a first set of ribozymes from shorter RNAs than one might have predicted, making the start of life more likely.

That is, in the chemisphere that might have existed, the two rate-limiting problems could have been solved simultaneously. A single early chemistry that provides globularity and catalytic power to short RNAs would have led to the “enzymatic” replication of template RNAs (e.g., genetics) that had been made initially without replication either by spontaneous condensation of monomers or in reactions catalyzed by other RNAs. We have used our own data about oligonucleotide globularity coupled with Harold B. White’s ideas about catalytic RNA (9–11) and a few simple oligonucleotide hairpin melting temperature (T_m_) experiments to address the problem inherent in short RNA molecules, and in doing so, perhaps, we have increased the plausibility of the RNA World Hypothesis.

## Discussion

Aptamers are single-stranded nucleic acid-based affinity reagents evolved from random libraries using SELEX (systematic evolution of ligands by exponential enrichment) (12, 13). Enhancement in the chemical diversity of these random nucleic acid libraries, even with one and the same modification at only one of the four bases (e.g., at a 5-position of a pyrimidine throughout the library), can dramatically improve both the success rate of DNA-based SELEX experiments and the binding affinities of the resulting aptamers (14–16). Crystal structures of these SOMAmer reagents (slow off-rate modified aptamers) bound to their cognate proteins make it clear that the pyrimidine adducts of SOMAmers form direct contacts with the protein and participate in shaping the binding interface, much like amino acid residues in proteins (17–21). As importantly, these crystal structures also show that the hydrophobic “side chains” of the modified bases participate in internal motifs that stabilize the folded structures (19).

**Fig. 1.**
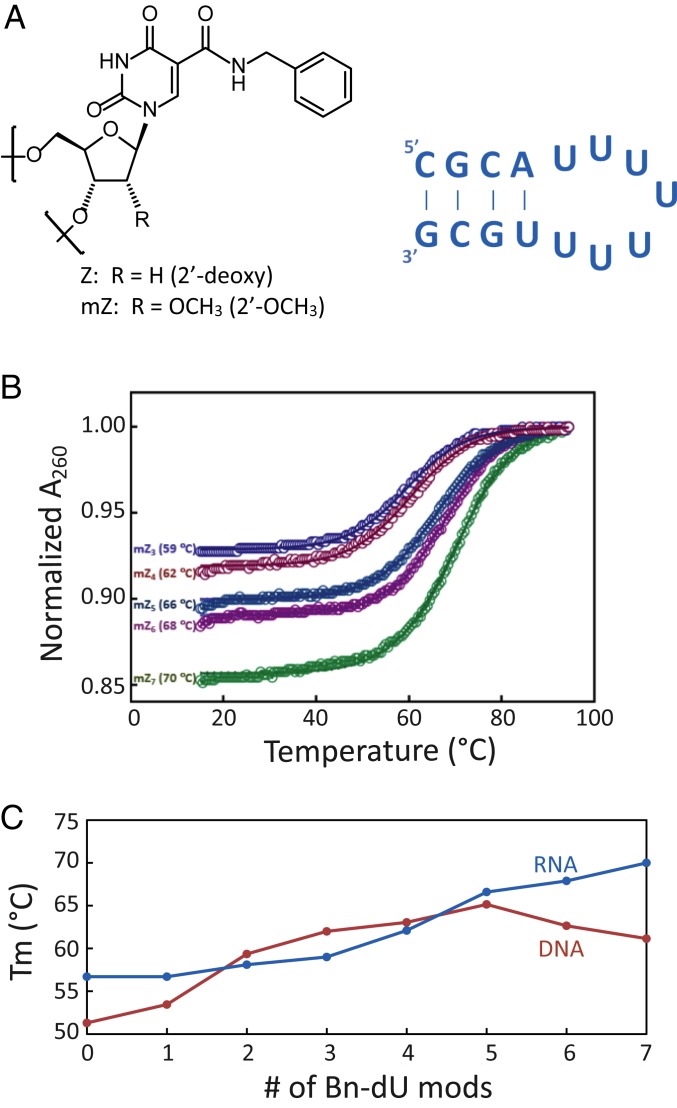
(*A*) Structure of the Bn-dU substitutions and the hairpin motif (RNA version) utilized in this study. (*B*) DNA and RNA series hairpin sequences and corresponding T_m_ values. (*C*) T_m_ values for each series as a function of the number of substitutions in the loop from zero to seven.

As a simple model of these interactions, we studied the effect of hydrophobic modifications on the thermodynamic stability of a short-hairpin structure in DNA and RNA (*SI Appendix*, Fig. S1). For DNA hairpins containing a stem region of four Watson–Crick base pairs and a loop of seven thymidine nucleotides (the T7 variant), the T_m_ increases steadily as up to five of these thymidines are replaced with 5-(*N*-benzylcarboxamide)-deoxyuridines (Bn-dU), with a total change of 14 °C (Fig. 1*C*). A very similar degree of stabilization is observed for the analogous RNA hairpin series in which the substituted modified nucleotides contain both 5-(*N*-benzylcarboxamide) base substitutions and 2′-*O*-methyl ribose substitutions (Fig. 1 *B* and *C*). As a control, we also included a sequence with a fully 2′-*O*-methyl–substituted loop to determine whether any observed stability gain is due to the base modifications, the ribose 2′-*O*-methyl modifications, or both. For this RNA series, the 2′-*O*-methyl groups contribute significantly to the T_m_ increase, showing that two vastly different types of hydrophobic modifications in the loop independently contribute to significant stabilization. It is possible that some of this stabilization results from the reduced conformational flexibility of the ribose ring associated with the presence of the 2′-*O*-methyl, which could be tested with NMR studies. In any event, the benzyl rings still provide a significant additional increase in T_m_.

The impact of the 2′-*O*-methyl groups alone is worthy of note because these modifications are present in modern RNA. These ribose modifications are known to increase the T_m_ values of RNA duplexes (22, 23) but to our knowledge, have not been shown to stabilize loop regions. T_m_ increases have been reported for 2′-*O*-methyl–modified RNA hairpins in the context of molecular beacons (24, 25), but in these examples, both the loop and stem regions were modified.

This modest thermodynamic dataset suggests that hairpin structure stabilization by the addition of hydrophobic modifications in the loop is significant for both DNA and RNA. The effect is surprisingly large. With regard to the RNA World, our experiment is a metaphor. There is little evidence regarding which modifications may have been present on early Earth, what concentrations of salts may have been present within relevant niches, or what degree of stabilization would be needed to facilitate an evolutionary transition toward globular structures that facilitate tertiary interactions required for catalysis.

With these simple preliminary experiments, we have asked and answered a narrow question. Will modified nucleobases stabilize a simple hairpin made of RNA or even DNA? A positive answer (which we obtained) suggests that an ordered loop structure can exist if simple hydrophobic adducts are substituted into hairpin loop regions. The data suggest that base modifications can create additional stable structures, which may facilitate the formation of pockets for substrate binding in single-stranded oligonucleotides.

We believe that we have reduced two questions to one, where the presence of the hydrophobic modifications facilitates solving both the length and catalysis problems simultaneously. This reduction in the number of independent variables can be thought of as an example of a reduction of dimensionality. This concept, while more commonly applied to machine learning and statistics, has been used in a biological context to describe solute/membrane receptor reaction rates mediated by two-dimensional diffusion along membrane surfaces (26–28) and by Berg and von Hippel (29) to explain the kinetic path through which the *lac* repressor protein searched *Escherichia coli* DNA for the single chromosomal *lac* operator. In the latter case, the needed three**-**dimensional collision between a protein and its binding site on a large genome was reduced to one-dimensional diffusion on the double-helical DNA genome.

Often, a reduction in dimensionality makes searches more efficient through the removal of an independent spatial dimension (30), such as in the examples cited above. In this evolutionary scenario, where the solutions to length and catalysis are no longer independent, the search space is reduced in an analogous way. The resulting faster search kinetics could have shortened the path to a breakout ribozyme, thus lending support to the RNA World Hypothesis.

We are aware that the benefits of this reduction of dimensionality are intimately tied to a complication associated with the need to distinguish one specific type of a modification (perhaps one endowed with particularly desirable catalytic properties) over other similar modifications (or no modifications) in order to preserve sequence information through multiple cycles of replication. The simplest solution to this problem is to propose that there was only one or at least a predominant modification in relevant prebiotic conditions so as to ensure uniform substitution throughout the RNA sequence that can be faithfully replicated (this idea would include individual pools of independently evolved precursors, each pool restricted to a single modified monomer). There is unsurprisingly an analogy with in vitro evolution. We use uniform modification at defined positions with our SELEX libraries precisely because multiple modifications at any one position, while having the desired effect of increasing chemical complexity in starting libraries, would dilute the number of exact copies of the original sequence with a precise pattern of modifications with each cycle of replication, thereby reducing the likelihood of copying the desired sequence for the next round of selection. The notion that only one or few modifications were present in prebiotic conditions seems unlikely. If there was a rich array of modifications, a mechanism would need to exist to selectively favor incorporation of certain modifications over others, which is also challenging. In the context of the RNA World, short functionally active sequence length enabled by modifications, as we are proposing here, would help with this problem but only to a limited extent. Another solution to this problem would be to propose that the introduction of specific modifications can be done by specific catalysts dedicated to such reactions (as it is done today with enzymes that modify transfer RNA [tRNA], for example), but this of course assumes that such entities already existed in prebiotic conditions. We do not provide a solution to this difficult problem here.

In our work in the development of nucleic acid-based affinity reagents, we have transitioned from RNA-based aptamers to DNA-based SOMAmer reagents (14–16). To date, we have made SOMAmers against more than 5,000 human proteins—a feat made possible by the expanded range of protein epitopes available for recognition by nucleic acids with pyrimidine 5-position modifications (14, 15). It was the structural insights gained from four cocrystal structures that made us wonder about their potential implications for the RNA World Hypothesis.

**Fig. 2.**
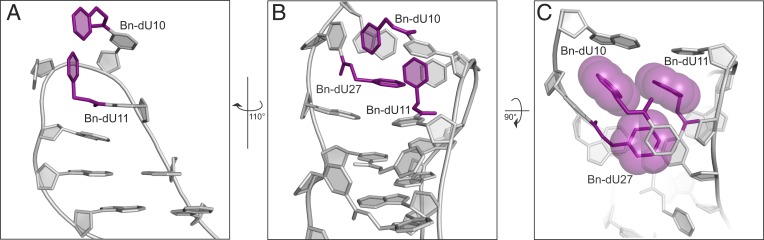
(*A*) Hydrophobic (benzyl) side chains at positions 10 and 11 within a single loop of the NGF SOMAmer (19). (*B*) Entire hydrophobic core including the benzyl side chain at position 27. (*C*) Space-filling model for the side chains within the hydrophobic core. Benzyl side chains are colored magenta and are designated Bn-dU.

The positions of some hydrophobic modifications in SOMAmer–protein cocrystals are shown in Fig. 2. A common theme observed in the cocrystal structures is the stabilization of nucleic acid ligands by the modified pyrimidines as illustrated by the SOMAmer that binds to nerve growth factor (NGF) (Fig. 2). Aside from two modified nucleotides in the loop at positions 10 and 11 (Fig. 2*A*), which resemble the simple model hairpin (Fig. 1*A*), another side chain from a distal part of the sequence (position 27) “intrudes” into this loop to form a hydrophobic cluster in an example of a long-range intramolecular interaction facilitated by base modifications (Fig. 2 *B* and *C*). In a fascinating example of plasticity of bases toward forming intramolecular contacts (and not just through hydrogen bonding), the benzyl side chains of residues 10 and 27 form reciprocal stacking interactions with the bases from the other nucleotide with a twist in the flexible 5-position linker to accommodate this interaction (Fig. 2*C*). We have observed such hydrophobic “zipper” motifs in another part of the NGF SOMAmer (19) as well as in another SOMAmer with a different (naphthyl) side chain (21). Together, these interactions make something quite complicated in three dimensions. Modifications lead to more intricate structures and stability: in other words, globular scaffolds with pockets akin to catalytic centers.

We have also observed some modification-driven intermolecular contacts for 5-position–modified sequences. This suggests that the hydrophobic modifications may provide yet another evolutionary component—solving what can be deemed “the proximity problem.” Formation of oligomers can be facilitated entropically by aligning to individual building blocks in space. Interaction of hydrophobic moieties in a hydrophilic environment could potentially provide the scaffolding for this alignment for intermolecular as well as intramolecular interactions, which is another mechanism for reducing the number of independent variables.

### Catalytic Power and Harold B. White.

As pointed out by Cermakian and Cedergren (31), early modifications to RNA catalysts could have provided a variety of potential benefits, including pH or thermal stability, expanded catalytic functionality, improved/expanded base-pairing capability, and expanded regulatory capabilities. Heuberger and coworkers (32) demonstrated experimentally that the 2-thiouridine modification stabilized A–U base pairs, potentially increasing the rate and fidelity of nonenzymatic templated RNA synthesis. In addition, the works of Benner et al. (33), Hirao (34), and Malyshev and Romesberg (35) demonstrated the ability of a variety of noncanonical bases to form stable base pairs, which supports the potential for modified bases to contribute to the stability of folded, hairpin-like structures in our proposed scenario.

The advantages of expanding the repertoire of functional groups to develop better catalysts have been duly recognized in the context of in vitro evolution from random libraries, and indeed, modified side chains that can serve as general acid or general base catalysts mimicking those observed in proteins have been used for this purpose (36–40). Finally, Rios and Tor (41) discussed the origin of the canonical bases and the fact that early selection pressures may have driven biology from the variety of structures present in the prebiotic soup toward the modern day structures. Although the dataset is not sufficiently large to generalize in our simple thermodynamic experiment, it seems striking that the addition of most modifications in the RNA (and DNA) loops yield additional stability. This suggests a potential incremental pathway for building catalytic activity along an evolutionary trajectory.

To examine how easily modifications could be accommodated into our model hairpin, we used RNAComposer (42) to generate the U7 stem loop (Fig. 1*A*) and then, manually added 5-(*N*-benzylcarboxamide) groups to each of the seven uridines using PyMOL Builder (43). This very simple model shows that it is possible for the modifications to be accommodated into the loop structure without evidence of bond strain and also serves as an example of potential side chain stacking interactions that could enhance stability.

Furthermore, with six rotational degrees of freedom in the phosphodiester backbone plus the rotational freedom of the glycosidic bond, even a relatively short seven-nucleotide sequence has a rich array of possible conformational states. Some of these conformations are compatible with the stem-loop structure, and most are not. The observation that seven fairly large hydrophobic side chains at the 5-position of each uridine can even fit into a loop (not a given) and at the same time, stabilize the stem-loop structure (Fig. 1*C*) suggests that the added steric bulk may constrain the repertoire of possible conformational states toward those compatible with stem-loop formation (entropic contribution). This stabilization via reduction in conformational entropy has been used to explain T_m_ increases in oligonucleotide duplexes containing locked nucleic acids (44) and T_m_ decreases for oligonucleotide duplexes with modified backbones containing increased numbers of linkage bonds (45). The presence of the aromatic rings may also contribute additional favorable stacking/hydrophobic interactions within the loop (enthalpic contribution).

Harold B. White (9) suggested that early nucleobases might have looked like (or included things like) the cofactors we know to improve catalysis by binding near/in the active sites of protein enzymes. He suggested that catalysis might have been available to primitive RNAs before modern biology took over because they contained chemical features or functional groups commonly found in cofactors that augmented their catalytic power. Those kinds of modifications stabilize complex oligonucleotide structures that allow shorter RNAs to be more functional (Fig. 2). More intricate structure encoded in short sequences enabled by modified side chains provides another quality needed for catalysis—enhanced potential for specificity—which is needed for preferential binding of transition state over ground state in chemical transformations. Such primordial, shorter molecules could have driven a primitive enzymology as the chemical Earth evolved toward replication and (later) protein-based enzymology, with the cofactors, according to White (9), as possible forensic remnants of the RNA-based catalysts that became the independent catalytic force they are today.

### Fossils of the RNA World (tRNAs and CUUCGG Hairpins).

Absent chemical fossils, we cannot know what the primordial cofactors were except that they could have enhanced both the catalytic power of early RNAs and their ability to function at shorter lengths. Two examples seem relevant to the discussion.

tRNAs have the highest densities of modification of any class of RNAs (46, 47). One might imagine that tRNAs could contain vestiges of early moments on Earth. As such, tRNA may represent a closer evolutionary relative to the early RNA before the separation of roles between catalysis and information storage. The fact that only a few modified nucleotides are observed in the same locations across the major phylogenic domains (48) may be a forensic hint to what the missing tRNA fossils would show.

Modified tRNAs have different stability of overall three-dimensional structure than unmodified tRNAs. Sampson and Uhlenbeck (49) showed that a tRNA with modifications has a cooperative melting curve rather than a sequential set of four helical melts from the four stems in the molecule. Modifications that exist today in tRNAs are directly involved in the structural integrity of the entire molecule, analogous to what we observe with modified nucleotides in our SOMAmer structures.

Furthermore, stable loops can be constructed from present day nucleotides. For example, CUUCGG and GAAA hairpin loops are known stabilizers of short stems. Tuerk and coworkers (50) showed that the extra stability from a UUCG loop is surprisingly large, of the same order of magnitude as we observed in this study (Fig. 1). We searched the bacteriophage T4 genome for CTTCGG sequences and found that, when this sequence is found within the sense strand, more than half of the time it is accompanied by flanking self-complementary regions that support hairpin formation. In contrast, this was not true for any of the occurrences within the antisense strand. This clearly suggests that stable loops remain functional today. As the RNA World moved away from modified bases (in White’s scenario), special loops might have replaced modified base-derived enhanced stabilities.

Clearly, only a few instances of unmodified sequences survived the test of time for hairpin loop stabilization. This is in striking contrast to our observation that most additions of a modified nucleotide to the loop resulted in an incremental increase in stability, strengthening the notion that modifications could have facilitated the early evolutionary development of catalytically capable nucleic acid structures.

### What Modified Bases Did the Chemical World Offer during the Transition from Chemistry to Biology?

The White paper on modified bases adds to the discussion of the function/catalysis problem, while the stabilization of a hairpin through modified loop bases potentially adds to the discussion of the length problem. The presence of prebiotic nucleobases modified at the 5-position has been proposed by Robertson and Miller (51). Uracil can react with formaldehyde to form 5-hydroxymethyluracil, which can then further react with phenol, tryptophan, imidazole, etc. to form amino acid analogs. This supports the possible early presence of modifications similar to those in our hairpins, and in addition, as proposed by these authors, it hints at the evolutionary path to modern proteins. However, the absence of true fossils leaves us in the dark—neither White nor anyone else knows what cofactors might have existed when the earliest chemically synthesized RNAs were formed. The White hypothesis—that the bases of RNA on the primordial Earth were themselves like the cofactors and thus, had an intrinsic capacity to be better catalysts than today’s RNAs—is not easily proven except by example.

## Conclusion

The RNA World Hypothesis faces the problem first declared by Leslie Orgel (52). How do early activated mononucleotides condense into polymers on the primitive Earth, providing the substrates and catalysts for a replicating system? Chemically derived oligonucleotide libraries must have folded into shapes that were capable of function: folded and functional RNAs require length (53). We have taken our work that was initially done for a different reason and presented it to shed light on the question that we posed earlier. How does one obtain catalytic RNAs that are short enough to exist in that chemical world and yet, catalytic enough to start our world of modern biology?

Joyce and Orgel (54) discussed the problem of emergence of self-replicating RNA molecules. They realized that movement toward an RNA world on a properly modeled prebiotic early Earth would have been continuously suppressed/thwarted by destructive reactions that shortened the RNAs. It was noted that many of the steps needed for formation of the nucleotides linkages do not proceed efficiently in prebiotic conditions. Joyce and Orgel (54) specifically suggested that the molecular biologist’s dream was “a magic catalyst” that could “convert the activated nucleotides to a random ensemble of polynucleotide sequences, a subset of which had the ability to replicate.” In a sense, all RNA World hypotheses are built around the idea that the breakout into true biology was a giant selection experiment using short oligonucleotides made by simple condensations in tiny pools of solvent as the starting molecules.

To that desire for a magic catalyst, we add, based only on our T_m_ data and inspiration from reading insightful papers, that cofactor-like primordial nucleobases simultaneously provide catalytic activity and diminish the oligonucleotide lengths needed to create intramolecular frameworks/active sites in RNAs. Our addition to the RNA World Hypothesis, dependent on Harold B. White’s brilliant idea of primordial cofactor-like RNA nucleobases, may provide a step toward the evolution and replication of early genomes and structured catalytic centers.

### What Would We Do Next?

David Perrin and coworkers (36, 55, 56) have shown that the catalytic efficiency of deoxyribozymes (in the absence of divalent cations) can be dramatically improved by utilizing random libraries with several concurrent base modifications that resemble side chains of amino acids found at the active sites of protein ribonuclease enzymes. In these examples, modification density clearly matters. With aptamers, we have recently shown that the use of libraries with two modified bases allows the identification of not only the highest-affinity ligands to a protein target but also, ligands in which high-affinity binding is encoded in short sequences (≤30 nucleotides) more frequently compared with libraries with a single modified base (57). To build on these results, one could choose the most catalytically competent pyrimidine adducts, put one on the 5-position of U and/or the other on the 5-position of C, and select catalytic RNAs in the standard ways. After identifying several catalytic RNAs of any length (e.g., 40 nucleotides to start), one would prepare libraries of decreasing length, reselect those catalysts, and at the same time, compare those catalysts with any that could be isolated in libraries absent the modifications. Our expectation is that catalytic modified RNAs would be far shorter than those derived from the unmodified libraries—that is the heart of our proposal. Should those experiments be successful, we would be able to say that our proposal has some merit. It would also be of interest to look at the impact of a variety of structural modifications and small molecule cofactors (such as Mg^2+^ and other divalent cations, spermidine, proflavine, etc.) on the stability of these hairpin structures and also on the outcome of these proposed selections.

However, the deeper question is not would our experiment be successful but how close to the chemical building blocks available in the chemical world would that experiment be. This problem seems to be at the heart of all experiments about the moment that chemistry became biology. As pointed out by Cairns-Smith (58) and Yarus (59), this is akin to trying to determine how a stone structure was built after the scaffolding that was used to aid construction is no longer present. Aiming at plausibility rather than certainty, as many other scientists who have thought deeply about this problem have realized, is a worthy goal.

We hope that people will tackle the length question systematically so that shorter modified RNAs could also be tested for catalytic breadth. The early Earth might have broken into biology from both short RNAs and broad catalytic opportunities.

Our proposal for the usefulness of hydrophobic modifications in early evolution creates additional questions with regard to specialization of function. For example, how is it possible that modifications were maintained throughout evolution along functional/structural paths, such as the folding of modern tRNAs, but did not persist with regard to storage of genetic information? In addition, what sequence of events is plausible for the loss of modifications along the heritability pathway? Considering the White hypothesis and the modern presence of modified nucleotides as cofactors, this may have correlated with the transition to the protein-based world. While these questions certainly cannot be intelligently addressed from the limited data presented here, we hope that these questions will be tackled as well.

## Materials and Methods

### DNA Synthesis and Purification.

Three of the DNA oligonucleotides were purchased from Integrated DNA Technologies. Other DNA oligonucleotides were synthesized in house via standard phosphoramidite methods with minor modifications to accommodate the 5-carboxamide modifications as described previously (60). The oligonucleotides were purified on a Waters 625 preparative high-performance liquid chromatography system (Waters 600 pumps, 486 detector, and a 600E controller) using strong anion exchange chromatography (mobile phase A = 10 mM tris(hydroxymethyl)aminomethane (Tris), 1 mM ethylenediaminetetraacetic acid (EDTA), pH 7.5; mobile phase B = 10 mM Tris, 1 mM EDTA, 2.0 M NaCl, pH 7.5; gradient 0 to 48% B over 20 min at room temperature) and a Source Q anion exchange column (6 mL; GE Healthcare). Purified samples were desalted using deionized water (18 MΩ, generated from an in-house system; Millipore Milli-Q Integral 10 system) and concentrated by ultrafiltration using a Pall Minimate tangential flow filtration system equipped with a 50-cm^2^, 1.0-K molecular weight cutoff polyethersulfone filter and a Cole Parmer Masterflex peristatic pump at a flow rate that kept the pressure below 30 psi. The purity of each oligonucleotide was determined by ion-pairing reverse-phase ultraperformance liquid chromatography as described previously (60), and the values were 81 to 93%.

### RNA Synthesis and Purification.

Standard and modified RNA oligonucleotides were synthesized in house via standard phosphoramidite methods using 2′-*tert*-Butyldimethylsilyl (TBDMS) protecting groups (61) on an ABI 3900 automated synthesizer using commercial reagents [rA(Bz) and rG(iBu) from Thermo Scientific, rC(Ac) and rU(CE) from Bioautomation, and 2′-OMe-rU from Proligo] and 2′-OMe-Bn-dU phosphoramidite, which was synthesized from 2′-OMe-rU (Proligo) as described previously (62). After initial cleavage and base deprotection with *t*-butyl amine:methanol:water (1:1:2) overnight at 37 °C, deprotection of the 2′-*O*-TBDMS ethers was accomplished using an ammonium fluoride/dimethylsulfoxide scheme (63).

The oligonucleotides were precipitated in ethanol. The resulting pellets were reconstituted in deionized water and then, purified using the same method described above for the DNA oligonucleotides. The purity of each oligonucleotide was determined by ion-pairing reverse-phase UPLC as described previously (60), and values were 86 to 96%.

### Optical Melting Experiments and T_m_ Calculations.

Optical melting experiments were done via absorbance at 260 nm in 1 M NaCl, 50 mM sodium phosphate, pH 7, as described previously (60). A subset of the heat-cooled SOMAmers was analyzed by polyacrylamide gel electrophoresis to verify that the melt data reflect an intramolecular transition. T_m_ values were calculated by two standard methods (64): 1) determining the maximum of the first derivative of the absorbance signal (dA_260_/dT) and 2) determining the temperature at which the fraction of molecules in the double-stranded state (θ) was equal to 0.5. Although T_m_ values calculated for each hairpin via the slope method were a few degrees higher than those obtained by determining the temperature at which θ = 0.5, the magnitude of the changes and the trends in the datasets were comparable. Only the T_m_ values calculated via θ = 0.5 are presented in Fig. 1*C* and *SI Appendix*, Fig. S1. For comparison and presentation, all data were normalized to an absorbance of 1.0 at 95 °C.

### Analysis of CUUCGG Sequences in the Enterobacteria Phage T4 Genome.

The genome sequence of Enterobacteria phage T4 was obtained from GenBank (NC_000866.4). We searched the genome for all occurrences of the sequence CTTCGG using Blat (65). We then extracted four bases upstream and downstream of each occurrence. We determined if each occurrence has the potential to form a hairpin if the four bases upstream of the CTTCGG sequence match the complement of the four bases downstream. We used the genome annotation to determine if each sequence overlapped with the coding sequence of a gene and if it was on the sense or antisense strand.

### Data Availability.

There are no data associated with this paper.

## Supplementary Material

Supplementary File
