# Supplementary material for: Modified nucleotides may have enhanced early RNA catalysis
Source: Proc Natl Acad Sci U S A. 2020 Mar 30;117(15):8236–42. doi: 10.1073/pnas.1809041117 (PMC7165471; doi:10.1073/pnas.1809041117)
Supplement: Supplementary File [file pnas.1809041117.sapp.pdf]

PNAS
www.pnas.org

Supplementary Information for

Modified Nucleotides May Have Enhanced Early RNA Catalysis

Steven K. Wolk, Wesley S. Mayfield, Amy D. Gelinas, David Astling, Jessica Guillot, Edward N. Brody, Nebojsa Janjic, Larry Gold[1]

SomaLogic, Inc., 2945 Wilderness Place, Boulder, CO 80301

[1]Address correspondence to: lgold@somalogic.com

This PDF file includes:

Supplementary text
Figures S1 to S2

www.pnas.org/cgi/doi/10.1073/pnas.1809041117

SUPPLEMENTARY INFORMATION

Results:

As a simple model of the stabilization of aptamer structures via hydrophobic interactions, we studied the effect of hydrophobic modifications at the 5-position of the uracil base on the thermodynamic stability of a short hairpin structure in DNA and RNA. (See Figures 1, S1, and S2.) For DNA hairpins containing a stem region of four Watson-Crick base pairs and a loop of 7 thymidine nucleotides (the T7 variant), the T_m increases steadily as up to 5 of these thymidines are replaced with 5-(N-benzylcarboxamide)-deoxyuridines (Z), with a total change of 14 °C (Figures 1C, S1 and S2), indicating that modifications greatly stabilize these short model hairpins. Further addition of modifications (6 or 7) results in a small decrease in the T_m, which may be due to steric hindrance within the loop region, requiring some interaction of the aromatic rings with bulk solvent. We did not attempt any further optimization for the optimum loop positions or other modifications.

Based on these results and the possible implications for the RNA world, we also synthesized an analogous series of RNA hairpins. In this series, we used ribonucleotides at all positions except at the 5-position-modified deoxyuridine positions, where we used 2'-O-methyl-substituted benzyl-modified nucleotides (mZ) due to the relative ease of its synthesis compared to the 2'-OH analogs. This is a reasonable approximation because both 2'-O-methyl and 2'-OH substituents favor the C3'-endo ribose conformation and therefore the A-form helix (1). Because this represents an additional modification, we included a control sequence with a fully 2'-O-methyl-substituted loop to determine whether any observed stability gain was due to the base modifications, the ribose 2'-O-methyl modifications, or both. The sequences and the measured T_m values are summarized in Figures 1, S1, and S2. Figure 1B clearly shows that the addition of each of these hydrophobic modifications results in a significant increase of T_m of the hairpin. The full substitution of 2'-O-methyl groups in the loop region yielded an increase of 9 °C, and the inclusion of additional 5-(N-benzylcarboxamide) groups yielded a total ΔT_m of 13 °C.

These data show that two different types of hydrophobic modifications (benzyls and methyl groups) on two different regions of the nucleic acid structure (bases and ribose groups, respectively) can both significantly stabilize a hairpin structure. In contrast to the DNA series, there was no drop in T_m as the 6th and 7th modifications were added, although the incremental increase in the T_m at this stage was lower.

SI References:

1.Guschlbauer W, Jankowski K (1980) Nucleoside conformation is determined by the electronegativity of the sugar substituent. Nucleic Acids Res 8(6):1421-33.

DNA Hairpin Series

name	# BndUs															Tm (°C)
Z_0	0	C	G	C	A	T	T	T	T	T	T	T	T	G	C	G	51.3
Z_1	1	C	G	C	A	T	T	T	Z	T	T	T	T	G	C	G	53.5
Z_2	2	C	G	C	A	T	Z	T	T	T	Z	T	T	G	C	G	59.4
Z_3	3	C	G	C	A	Z	T	T	Z	T	T	Z	T	G	C	G	62.0
Z_4	4	C	G	C	A	Z	T	Z	T	Z	T	Z	T	G	C	G	63.1
Z_5	5	C	G	C	A	Z	Z	T	Z	T	Z	Z	T	G	C	G	65.2
Z_6	6	C	G	C	A	Z	Z	Z	T	Z	Z	Z	T	G	C	G	62.7
Z_7	7	C	G	C	A	Z	Z	Z	Z	Z	Z	Z	T	G	C	G	61.2

RNA Hairpin Series

name	# mBndUs																Tm (°C)
U_7	0	rC	rG	rC	rA	U	U	U	U	U	U	U	U	rG	rC	rG	56.7
mU_7	0	rC	rG	rC	rA	mU	mU	mU	mU	mU	mU	mU	U	rG	rC	rG	65.6
mZ_1	1	rC	rG	rC	rA	U	U	U	mZ	U	U	U	U	rG	rC	rG	56.7
mZ_2	2	rC	rG	rC	rA	U	mZ	U	U	U	mZ	U	U	rG	rC	rG	58.1
mZ_3	3	rC	rG	rC	rA	mZ	U	U	mZ	U	U	mZ	U	rG	rC	rG	59.0
mZ_4	4	rC	rG	rC	rA	mZ	U	mZ	U	mZ	U	mZ	U	rG	rC	rG	62.1
mZ_5	5	rC	rG	rC	rA	mZ	mZ	U	mZ	U	mZ	mZ	U	rG	rC	rG	66.6
mZ_6	6	rC	rG	rC	rA	mZ	mZ	mZ	U	mZ	mZ	mZ	U	rG	rC	rG	67.9
mZ_7	7	rC	rG	rC	rA	mZ	mZ	mZ	mZ	mZ	mZ	mZ	U	rG	rC	rG	70.0

Fig. S1. DNA and RNA series hairpin sequences and corresponding T_ms.

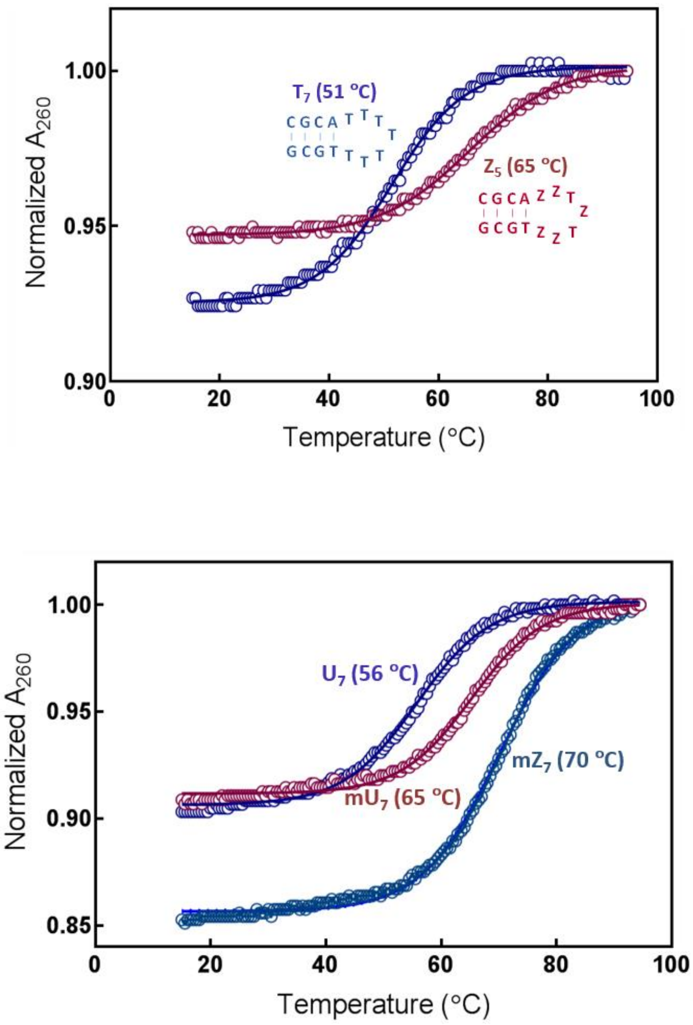

Fig. S2. Example optical melting curves demonstrating the impact of 5-(N-benzylcarboxamide)-deoxyuridine substitutions on the melting temperature of DNA and RNA hairpin loops; (A) comparison of the melting curves of the T_7 and Z_5 variants in the DNA series; (B) comparison of the melting curves of the U_7, mU_7, and mZ_7 variants in the RNA series.
